# The Relationship between Psychological Distress during the Second Wave Lockdown of COVID-19 and Emotional Eating in Italian Young Adults: The Mediating Role of Emotional Dysregulation

**DOI:** 10.3390/jpm11060569

**Published:** 2021-06-17

**Authors:** Anna Guerrini Usubini, Roberto Cattivelli, Giorgia Varallo, Gianluca Castelnuovo, Enrico Molinari, Emanuele Maria Giusti, Giada Pietrabissa, Tommaso Manari, Maria Filosa, Christian Franceschini, Alessandro Musetti

**Affiliations:** 1 Istituto Auxologico Italiano IRCCS, Psychology Research Laboratory, 20145 Milan, Italy; u.guerrini@auxologico.it (A.G.U.); r.cattivelli@auxologico.it (R.C.); g.varallo@auxologico.it (G.V.); gianluca.castelnuovo@auxologico.it (G.C.); molinari@auxologico.it (E.M.); g.pietrabissa@auxologico.it (G.P.); 2 Department of Psychology, Catholic University of Milan, 20123 Milan, Italy; 3 Department of Humanities, Social Sciences and Cultural Industries, University of Parma, 43121 Parma, Italy; tommaso.manari@unipr.it (T.M.); alessandro.musetti@unipr.it (A.M.); 4 Department of Medicine and Surgery, University of Parma, 43121 Parma, Italy; filosa.maria@hotmail.it (M.F.); Christian.franceschini@unipr.it (C.F.)

**Keywords:** COVID-19, young adults, social isolation, psychological distress, emotional eating, emotional dysregulation

## Abstract

This cross-sectional study aims to investigate the impact of psychological distress experienced during the second wave of the COVID-19 pandemic on emotional eating and to assess the mediating role of emotional dysregulation in a sample of Italian young adults (20–35). A total of 437 participants provided demographical data and were assessed using the Depression Anxiety Stress Scale, the Difficulties in Emotion Regulation Scale, and the Emotional Eating subscale of the Dutch Eating Behavior Questionnaire. Correlational analyses were performed to assess the relationship between continuous variables, while ANOVA was conducted to detect differences between males and females for emotional eating. To assess whether demographic and clinical data predicted emotional eating, hierarchical linear regression was performed. Then, a mediation analysis was conducted to assess whether emotional dysregulation was a mediator between psychological distress and emotional eating. Emotional eating was associated with psychological distress and emotional dysregulation. Moreover, higher levels of emotional eating were found in females than in males. Predictors of emotional eating were sex, psychological distress, and emotional dysregulation. Mediation analyses showed that the indirect effect of psychological distress on emotional eating through emotional dysregulation was significant (b = 0.0069; SE = 0.0024; CI = 0.0024–0.0118), confirming that the relationship between psychological distress and emotional eating was mediated by emotional dysregulation, controlling for sex. The model explained 26.8% (*R*^2^ = 0.2680) of the variance. These findings may help to plan and develop psychological interventions aimed at addressing emotional eating in young adults by targeting emotional dysregulation.

## 1. Introduction

Coronavirus disease 19 (COVID-19), a new form of severe acute respiratory syndrome coronavirus 2 (SARS-CoV-2), was firstly identified in Wuhan City (China) in December 2019. Since then, COVID-19 has rapidly spread throughout China and has quickly become a global health concern. After China, Italy was one of the first countries in which COVID-19 spread. The first infections were recorded at the end of January 2020, and over the next few months the number of cases grew exponentially. Facing an increasing number of cases, the Italian government has implemented extraordinary preventive measures based on social distancing, limitation of movement and physical interaction, and unprecedented quarantine measures. Citizens were asked to isolate themselves and were not allowed to leave their homes except for well-documented reasons. Non-essential activities and schools were closed, and most workers were restricted to working from home or stopping work. On 11 March 2020, Italy was locked down. This extreme measure was adopted until 4 May 2020. Then, a subsequent reduction in case numbers allowed the Italian government to reduce the imposed containment measures in summer. Unfortunately, after a period of decreased case numbers, a second wave of COVID-19 began, and Italy once more faced a series of restrictive measures, even if there was different severity in different areas.

Although it was necessary to halt the contagion curve, the prolonged restrictive measures harmed the physical and mental health of the Italian general population, generating a variety of psychological problems. Recent studies reported increased anxiety and depressive symptoms, post-traumatic stress, digestive symptoms [1,2], compulsive and addictive behaviors [3,4,5], and poor sleep quality [6,7] in the Italian population during the lockdown.

The negative impact of the COVID-19 pandemic and the associated restrictive measures was particularly marked among specific populations, including young adults who typically report negative psychological consequences during health emergencies [8,9]. Young adults reported negative psychological effects related to the pandemic [7,8,9]. In Italy, a study [10] showed that during the first four weeks of lockdown (from 16 March to 16 April, 2020), Italian young adults (19–29) reported an increase in internalizing problems, including depression, anxiety, withdrawal, and somatic complaints, and externalizing problems, such as aggressive and rule-breaking behaviors. Conversely, the perception of personal strengths decreased.

Stress, anxiety, and depression due to the COVID-19 pandemic and subsequent restrictive measures had a negative impact on eating behaviors [9]. In a recent study aimed at exploring changes in eating habits during the lockdown using an Italian sample, individuals reported eating more than usual and eating unhealthy food. Participants also attributed changes in their eating habits to increased anxiety caused by COVID-19 and subsequent lockdowns [10]. In another Italian cross-sectional study, the authors found that participants reported eating in response to negative feelings of anxiety and increasing their food intake [11].

This scenario reflects emotional eating. Emotional eating is defined as “the tendency to eat in response to negative emotions” [12]. Emotional eating could be problematic for both physical and psychological health since it has been associated with consuming unhealthy food and, therefore, weight gain, as well as with poorer psychological well-being, depression [13], and eating disorders [14,15]. Psychological distress, in particular depression and anxiety, was found to be a risk factor for the onset of eating disorders. For instance, it has been found that individuals with low mood engage in disordered eating behaviors to feel comfort from aversive emotional states [16]. Even though it is well recognized that emotional eating is triggered by psychological distress and negative mood, the mechanisms underlying this relationship are yet to be addressed [17].

In the literature, one of the key factors associated with emotional eating is emotional dysregulation [18]. Gratz and Roemer [19] proposed a multidimensional conceptualization of emotional regulation that included the awareness and acceptance of experienced emotions and the control of impulsive behaviors when experiencing negative emotions in order to behave in accordance with desired goals. In addition, it included the ability to use appropriate emotional regulation strategies to flexibly modulate emotional responses to situations. Based on this model, emotional regulation strategies allow individuals to act in accordance with personal goals, even in the presence of negative emotions, while controlling impulsive behaviors. On the other hand, emotional eating is generally used to regulate negative feelings when emotional regulation abilities are lacking. In light of this model, a recent study found that psychological distress, in particular anxiety, was related to “drunkorexia”—an eating disorder that is characterized by indulging in weight control behaviors in relation to drinking alcohol—in the presence of higher levels of emotional dysregulation in a sample of non-clinical adolescents [20]. On the basis of the same model of emotion dysregulation, Squires and colleagues [21] found a positive and significant correlation between psychological distress and emotional dysregulation. These findings were also reported in previous studies [22,23,24].

Emotional dysregulation was found to be an underlying mechanism of emotional eating. McAtamney and colleagues [25] recently explored the mediating role of emotional dysregulation between the difficulty in describing feelings (alexithymia) and emotional eating in a sample of 136 participants recruited from the general population in the United Kingdom in July 2020 after a period of lockdown. Results showed an indirect effect of emotional dysregulation by which difficulties in identifying and describing emotions predicted emotional eating. By outlining the mechanism underpinning emotional eating, findings from that study increased awareness about how eating behaviors changed in the context of the pandemic.

To the best of the authors’ knowledge, no studies have been carried out to assess emotional eating in Italian young adults during the COVID-19 pandemic. 

Therefore, the current study aimed to explore the relationship between psychological distress related to the second COVID-19 lockdown and emotional eating. Moreover, the second aim of the study was to investigate the mediating role of emotional dysregulation in the link between psychological distress and emotional eating. 

In particular, we hypothesized that the relationships between psychological distress and emotional dysregulation and between emotional dysregulation and emotional eating would be significant. In addition, we hypothesized that there would be a significant relationship between psychological distress and emotional eating. Finally, we hypothesized that the relationship between psychological distress and emotional eating would be mediated by emotional dysregulation. 

## 2. Materials and Methods

### 2.1. Participants and Procedures

This cross-sectional study is part of a larger research project called “Effects of the second wave COVID-19 on general population: sleep quality and hyperconnectivity”. Data were collected from 1 December 2020 to 31 January 2021 during the second wave of COVID-19 in Italy. A convenience sample of 437 Italian young adults completed an anonymous online survey via the Microsoft Azure platform after providing written informed consent.

Inclusion criteria were a) age between 20 and 35 years, b) Italian mother tongue, and c) living in Italy during the second wave of COVID-19 lockdown. 

The Ethical Committee of the Center for Research and Psychological Intervention (CERIP) of the University of Messina approved the study (protocol number: 17758). All procedures were conducted in accordance with the Declaration of Helsinki and its later advancements. 

### 2.2. Measures

The survey involved demographical and clinical measures. Demographical data included sex, age, nationality, work status, marital status, weight, and height. Body mass index (BMI = kg/m^2^) was obtained by dividing weight expressed in kilograms by the square of height in meters. To assess clinical variables, we used the Italian validated questionnaires discussed below.

Psychological distress. The *Depression Anxiety Stress Scale* (DASS-21) [26] was administered to measure psychological distress. It is a self-report questionnaire composed of 21 items, rated on a 4-point Likert scale, ranging from 0 to 3, which explores three subscales: depression, anxiety, and stress. The total score of DASS-21 was used as a measure of psychological distress. We used the Italian version validated by Bottesi and colleagues [27] that showed good psychometric properties (Cronbach’s alpha values of subscales ranged from 0.83 to 0.91. The Cronbach’s alpha of the total score was = 0.92. In our sample, the Cronbach’s alpha of the total score was excellent (Cronbach’s alpha = 0.94).

Emotional dysregulation. The Difficulties in Emotion Regulation Scale (DERS) [19] was administered to assess difficulties in emotional regulation. This is a self-report questionnaire consisting of 36 items, rated on a 5-point Likert scale, ranging from 1 (almost never) to 5 (almost always), which explores the following subscales: non-acceptance of negative emotions, inability to undertake purposeful behavior when experiencing negative emotions, difficulty in controlling impulsive behavior when experiencing negative emotions, limited access to emotion regulation strategies that are considered effective, lack of awareness of one’s emotions, and lack of understanding of the nature of one’s emotional responses. We used the Italian version validated by Giromini and colleagues [28] that showed good psychometric properties. The Cronbach’s alpha of the total score was 0.92. In our sample, the Cronbach’s alpha of the total score was excellent (Cronbach’s alpha = 0.90)

Emotional eating. The Emotional Eating subscale of the Dutch Eating Behavior Questionnaire (EE_DEBQ) [15] was administered to assess emotional eating. The DEBQ is a self-report questionnaire used to assess eating behaviors. The Emotional Eating subscale consists of 13 items, rated on a 5-step Likert scale, ranging from 0 (never) to 4 (almost always). We used the Italian version validated by Dakanalis and colleagues [29] that showed good psychometric properties (Cronbach’s alpha = 0.97). In our sample, the Cronbach’s alpha of the subscale was excellent (Cronbach’s alpha = 0.95) 

### 2.3. Statistical Analysis 

Frequencies and percentages for categorical variables and means and standard deviations for continuous variables were computed. To assess normal distribution of variables, skewness and kurtosis were evaluated. Parameters outside the limit of +1.5/−1.5 range were considered an index of non-normality. Bivariate Pearson’s correlations were calculated to assess the correlations between all the continuous demographical (age and BMI) and clinical (psychological distress, emotional dysregulation, and emotional eating) variables. Univariate analysis of variance (ANOVA) was performed to assess whether males and females differed in emotional eating. A hierarchical linear regression model was used to determine which factors were predictors of emotional eating. Mediation analysis was performed to assess the mediating role of emotional dysregulation in the relationship between psychological distress and emotional eating using Model 4 of PROCESS Macro for SPSS [30]. An estimation of the indirect effect was obtained using the bias-corrected bootstrapping method (5,000 samples). Then, 95% bias-corrected confidence intervals (BC-CIs) were calculated to determine the significance of the mean indirect effects. The indirect effect was considered statistically significant at *p* < 0.05 when 95% BC-CIs did not include zero.

Analyses were performed using Jamovi (1.6.15) and IBM Statistical Package for the Social Sciences SPSS version 26 (Armonk, NY: IBM Corp). 

## 3. Results

### 3.1. Descriptive Statistics of the Sample and Relations to Emotional Eating 

**Figure 1:**
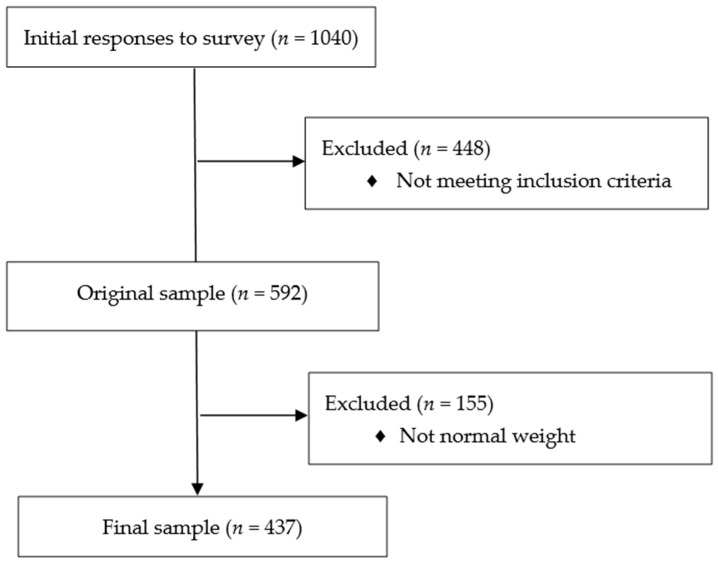
Flow chart. Bivariate correlations revealed that the demographic variables of age and BMI were not significantly correlated with emotional eating. As far as psychological distress is concerned, there was a significant and positive correlation between the total score of DASS-21 (r = 0.395; *p* < 0.001) and emotional eating. Emotional dysregulation, assessed with the total score of DERS, was significantly and positively associated with emotional eating (r = 0.348; *p* < 0.001). All correlations are presented in Table 2.

After subjects who did not meet inclusion criteria were excluded, 592 subjects filled out the online survey. In order to have a normal-weight sample, we excluded participants with BMI less than 18.5 and more than 25 (WHO, 2000). The final sample was composed of 437 participants. There were 213 (48.7%) males and 224 (51.3%) females; the mean age was 25.2 (SD = 5.12). Most of participants were Italian (97.3%), had a high school degree (68.2%), were students (38%), and were single (69.8%). Missing data were less than 5% and so were considered negligible [29]. The descriptive statistics of the sample are presented in Table 1. A flow chart of the recruitment of the sample is shown in Figure 1.

ANOVA test results indicated that there was a significant difference between males and females in emotional eating (F(1,413) = 64.84; *p* < 0.001). Females reported greater emotional eating (M = 2.38; SD = 0.94) than males did (M = 1.74; SD = 0.71).

### 3.2. Predictors of Emotional Eating 

To assess whether demographic and clinical variables were predictors of emotional eating, a multiple hierarchical linear regression model was performed. The model was built to detect the effect of psychological distress and emotional dysregulation on emotional eating controlling for sex, the only demographical variable related to emotional eating. Sex was added as a control variable at the first block; the total score of DASS-21 at the second block; and the total score of DERS at the third. 

The first model accounted for a significant amount of variance in emotional eating (*R*^2^ = 0.13; *p* < 0.001; F(1434) = 64.1; *p* < 0.001). Then, the total score of DASS-21 was added at the second block. The model explained 25% of the variance for emotional eating (*R*^2^ = 0.25; *p* < 0.001; F(2433) = 72.4; *p* < 0.001). Finally, the total score of DERS was added at the third block. The final model accounted for 27% of the variance for emotional eating (*R*^2^ = 0.27; *p* < 0.001; F(3432) = 52.7; *p* < 0.001). The results are presented in Table 3. 

### 3.3. Mediation Analysis

To assess the hypothesis that psychological distress might influence emotional eating through emotional dysregulation, mediation analysis was performed. The independent variable was psychological distress, the outcome variable was emotional eating, and the mediator was emotional dysregulation. In order to take the impact of sex into account, it was added as a covariate in the model.

The results showed that the total effect of psychological distress on emotional eating was significant (b = 0.0248; SE = 0.0030; *p* < 0.001; CI = 0.0190–0.0306). In addition, with the inclusion of the mediator, the direct effect of psychological distress on emotional eating was significant (b = 0.0179; SE = 0.0036; *p* < 0.001; CI = 0.0108–0.0250). Again, the indirect effect of psychological distress on emotional eating through emotional dysregulation was found to be significant (b = 0.0069; SE = 0.0024; CI = 0.0024–0.0118). The results also suggest that the indirect mediated effect accounted for 26.8% (*R*^2^ = 0.2680) of the variance. This evidence suggests that the relationship between psychological distress and emotional eating is partially mediated by emotional dysregulation.

**Figure 2:**
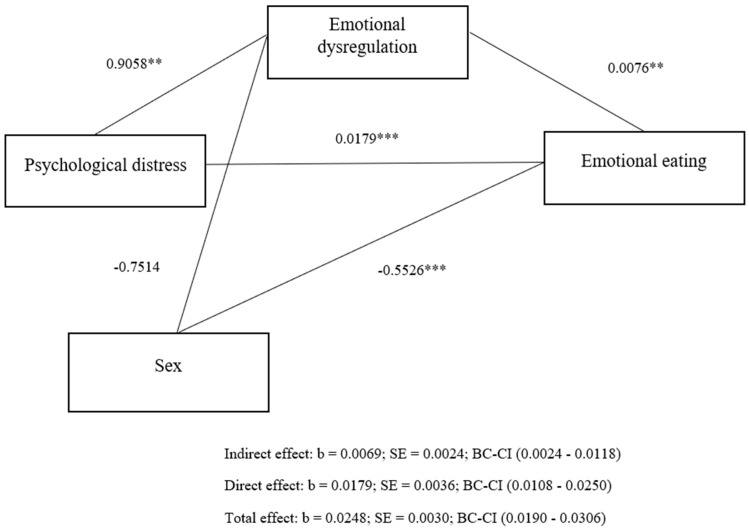
Mediation model for the relationship between psychological distress, emotional, and emotional eating, with sex as a covariate. Note: DASS-21: Depression Anxiety and Stress scale; DERS: Difficulties in Emotional Regulation Scale; EE_DEBQ: Emotional Eating subscale of the Dutch Eating Behavior Questionnaire; ** *p* < 0.05, *** *p* < 0.001.

The model is presented in Figure 2.

## 4. Discussion

The restrictive measures introduced to counter the COVID-19 outbreak dramatically affected the physical and psychological health of the Italian population. Among others, Italian young adults were seriously harmed by the COVID-19 outbreak [11], with an increase in both internalizing and externalizing problems during the first lockdown. 

The pandemic strongly affected the daily habits and changed the lifestyle of the Italian population [14]. In particular, there was an increase in disordered eating, including emotional eating, provoked by the COVID-19 outbreak [12,13,14]. However, the underlying mechanism explaining the relationship between psychological distress related to the pandemic and emotional eating was unclear.

This study was conceived to explore the impact of psychological distress due to the lockdown measures on emotional eating in a sample of Italian young adults by assessing the mediating role of emotional dysregulation. 

As hypothesized, psychological distress during the second wave of lockdown was related to emotional eating, and this relationship was partially mediated by emotional dysregulation. Specifically, according to our results, emotional eating was found to be related to emotional dysregulation and psychological distress, particularly depression, anxiety, and stress. Moreover, higher levels of emotional eating were reported in women than in men. These findings were in line with previous studies illustrating that emotional eating was triggered by psychological distress, including anxiety, depression, stress, and emotional dysregulation [31,32,33,34,35]. In addition, the difference we identified between males and females in regard to emotional eating behavior was supported by previous findings in the literature [15,29]. Sex differences in eating may be due to several factors, including medical and psychological differences between males and females. In particular, females generally show higher levels of anxiety, depression, and stress than men, as well as higher body dissatisfaction, all of which correlate with disordered eating [36]. 

We did not find significant associations between emotional eating and age or emotional eating and BMI. It could be hypothesized that these inconsistent relationships-that were previously demonstrated [29]-were most likely due to the composition of our sample, which included only young adults with normal weight. 

Our findings are consistent with the affect regulation model, which suggests that maladaptive behaviors, such as eating in response to negative feelings, function as an attempt to alleviate negative emotions [36]. It also extends the model using a sample of Italian young adults dealing with the psychological consequences of the COVID-19 pandemic and the related restrictive measures. 

From a clinical point of view, our results have important implications. The global pandemic requires researchers and clinicians not only to assess and monitor the psychological implications of the pandemic but also to plan and develop efficient psychological interventions to take care of citizens’ mental health, with a particular emphasis on high-risk groups, such as young adults. By assessing the role of emotional dysregulation on the link between psychological distress and emotional eating, the current study could inform interventions aimed at mitigating the negative effects of COVID-19 on eating habits by promoting emotional regulation strategies. Such interventions may help individuals to notice and regulate their internal states without using food to deal with their emotions.

Several limitations of the present study must be considered. Firstly, the cross-sectional nature of this study does not allow us to carry out causal explanations of relations among variables. Control group, manipulation, and longitudinal measures are lacking. Moreover, additional variables that were not taken into account in this study could play a role in emotional eating, such as alexithymia [25,37]. However, in the present study, possible confounder variables, such as age and BMI, were addressed. Secondly, all the measurements were self-reported and could therefore be affected by bias. Another limitation is related to the sample. This study used a convenience sample, a type of non-probability sample that confers many advantages, such as a quick and inexpensive data collection, as well as disadvantages, such as selection bias and reduced representativity. Future replications of the study would be helpful to reduce bias in convenience sampling by using probability sampling. Future studies should examine the role of other variables that could play a key role in influencing emotional eating. In addition, future research could consider samples of under- or over-weight young adults to extend the findings. 

## Figures and Tables

**Table 1:** Descriptive statistics of the sample.

	*N* (%)	Mean ± SD	Range
Sex			
Male	213 (48.7%)		
Female	224 (51.3%)		
Age (in years)		25.2 ± (5.12)	20–35
BMI (Kg/m2)		21.9 ± (1.73)	18.5–25
Nationality			
Italian	425 (97.3%)		
Non-Italian	12 (2.7%)		
Educational level			
Primary school	0 (0%)		
Secondary school	11 (2.5%)		
Higher school	298 (68.2%)		
Bachelor’s degree	120 (27.5%)		
Master’s degree	8 (1.8%)		
Marital status			
Single	305 (69.8%)		
Married	131 (30%)		
Divorced	1 (0.2%)		
Work status			
Student	166 (38%)		
Student and employed	82 (18.8%)		
Employed	161 (36.8%)		
Unemployed	16 (3.7%)		
Other	12 (2.7%)		
DASS-21		40.5 ± (12.8)	21–80
DERS		89.7 ± (19.5)	36–156
EE_DEBQ		2.07 ± (0.9)	1–5

Note: BMI: body mass index; DASS-21: Depression Anxiety and Stress scale; DERS: Difficulties in Emotional Regulation Scale; EE_DEBQ: Emotional Eating subscale of the Dutch Eating Behavior Questionnaire.

**Table 2:** Relationships between all the variables of interest.

	Age	BMI	DASS-21	DERS	EE_DEBQ	F	*p*
Age							
BMI	0.196 ***						
DASS-21	−0.160 ***	−0.078					
DERS	−0.137 ***	−0.086	0.580 ***				
EE_DEBQ	−0.074	0.024	0.395 ***	0.348 ***			
Sex						64.84	<0.001

Note: BMI: body mass index; DASS-21: Depression Anxiety and Stress scale; DERS: Difficulties in Emotional Regulation Scale; EE_DEBQ: Emotional Eating subscale of the Dutch Eating Behavior Questionnaire;*** *p* < 0.001.

**Table 3:** Hierarchical multiple regression with emotional eating as a dependent variable.

	Predictor	*R* ^2^	*Adj R* ^2^	F	*p*	B	SE B	β	*p*
Model 1		0.129	0.127	64.1	<0.001				
	Constant					2.385	0.0561		<0.001
	Sex					−0.644	0.0805	−0.717	<0.001
Model 2		0.251	0.247	72.4	<0.001				
	Constant					1.3403	0.13488		<0.001
	Sex					−0.5583	0.07540	−0.621	<0.001
	DASS-21					0.0248	0.00295	0.353	<0.001
Model 3		0.268	0.263	52.7	<0.001				
	Constant					0.93462	0.18392		<0.001
	Sex					−0.55264	0.07463	−0.615	<0.001
	DASS-21					0.01789	0.00362	0.255	<0.001
	DERS					0.00760	0.00237	0.165	0.001

Note: BMI: body mass index; DASS-21: Depression Anxiety and Stress scale; DERS: Difficulties in Emotional Regulation Scale; EE_DEBQ: Emotional Eating subscale of the Dutch Eating Behavior Questionnaire.

## Data Availability

The collected in this study are available on request from the author A.G.U. The data are not publicly available due to privacy/ethical restrictions.
